# Semi‐automated vertex placement for lattice radiotherapy and dosimetric verification using 3D polymer gel dosimetry

**DOI:** 10.1002/acm2.14489

**Published:** 2024-08-26

**Authors:** Tenzin Kunkyab, Anthony Magliari, Andrew Jirasek, Benjamin Mou, Derek Hyde

**Affiliations:** ^1^ Department of Computer Science, Mathematics, Physics and Statistics The University of British Columbia Okanagan Kelowna British Columbia Canada; ^2^ BC Cancer Kelowna British Columbia Canada; ^3^ Varian Medical Systems Palo Alto California USA; ^4^ Department of Surgery The University of British Columbia Vancouver British Columbia Canada

**Keywords:** 3D dosimetry, 3D polymer gel dosimetry, cone‐beam CT, lattice radiotherapy, spatially fractionated radiotherapy

## Abstract

**Purpose:**

To evaluate the feasibility of an open‐source, semi‐automated, and reproducible vertex placement tool to improve the efficiency of lattice radiotherapy (LRT) planning. We used polymer gel dosimetry with a Cone Beam CT (CBCT) readout to commission this LRT technique.

**Material and methods:**

We generated a volumetric modulated arc therapy (VMAT)‐based LRT plan on a 2 L NIPAM polymer gel dosimeter using our Eclipse Acuros version 15.6 AcurosXB beam model, and also recalculated the plan with a pre‐clinical Acuros v18.0 dose calculation algorithm with the enhanced leaf modelling (ELM). With the assistance of the MAAS‐SFRThelper software, a lattice vertex diameter of 1.5 cm and center‐to‐center spacing of 3 cm were used to place the spheres in a hexagonal, closed packed structure. The verification plan included four gantry arcs with 15°, 345°, 75°, 105° collimator angles. The spheres were prescribed 20 Gy to 50% of their combined volume. The 6 MV Flattening Filter Free beam energy was used to deliver the verification plan. The dosimetric accuracy of the LRT delivery was evaluated with 1D dose profiles, 2D isodose maps, and a 3D global gamma analysis.

**Results:**

Qualitative comparisons between the 1D dose profiles of the Eclipse plan and measured gel showed good consistency at the prescription dose mark. The average diameter measured 13.3 ± 0.2 mm (gel for v15.6), 12.6 mm (v15.6 plan), 13.1 ± 0.2 mm (gel for v18.0), and 12.3 mm (v18.0 plan). 3D gamma analysis showed that all gamma pass percent were > 95% except at 1% and 2% at the 1 mm distance to agreement criteria.

**Conclusion:**

This study presents a novel application of gel dosimetry in verifying the dosimetric accuracy of LRT, achieving excellent 3D gamma results. The treatment planning was facilitated by publicly available software that automatically placed the vertices for consistency and efficiency.

## 1 INTRODUCTION

Lattice radiotherapy (LRT) is a type of spatially fractionated radiotherapy (SFRT), developed by Wu et al. in 2010.^1^ The treatment planning requires multiple high‐dose spheres, called vertices, separated by a specified distance to achieve a high peak‐to‐valley dose difference.^2^ This process ultimately results in a heterogenous dose distribution within the irradiated volume, which deviates from the currently accepted paradigm of treating an entire tumor with homogenous dose distribution. Part of the LRT treatment planning process involves optimizing the placement of the spheres to limit the dose in the periphery of the tumor and minimize toxicities to adjacent surrounding normal tissues.^3^

LRT has been shown to be clinically useful in patients with large and unresectable tumors, resulting in high local control rates and low‐grade toxicity at patient follow‐up.^4^, ^5^ To date, most of the clinical data that demonstrates efficacy of the LRT technique are limited to individual case reports and series. Two studies by Amendola et al. implemented LRT in 30 advanced gynecological and 10 advanced non‐small‐cell lung cancer (NSCLC) patients.^4^, ^6^, ^7^ High local control rates, excellent clinical and imaging‐based follow‐up results were reported in the gynecological study, even after 4 years.^4^ In the lung cancer patients, LRT exhibited statistically significant tumor shrinkage of 42%, with improved long‐term average survival rate of 22 months.^6^ A more recent phase 1 trial by Duriseti et al. reported that LRT is a safe form of palliative treatment for large tumours, exhibiting significant volume shrinkage and no significant toxicity or deterioration in quality of life.^8^ Hypothesized radiobiological effects surrounding LRT include bystander‐like effect (local) and abscopal effect (distant).^3^, ^9^, ^10^

Clinical adoption of LRT is challenging in terms of planning and dosimetric verification. The manual placement of the vertices is a laborious process^11^ and bulkier tumor volumes require a large number of localized vertices, increasing the workload for the treatment planner. Second, there are dosimetry challenges associated with the heterogeneous dose distribution delivered utilizing the LRT technique. The complex spatial arrangement of the vertices, in 3D space, is difficult to verify with the traditional point dose and planar dose measurement techniques that are commonly used in many radiotherapy centers. Therefore, an accurate dosimetric and geometric verification of the high and low‐dose regions inside the tumor volume is necessary. For widescale clinical implementation of LRT, overcoming these challenges associated with LRT planning and dosimetric verification are essential.

3D polymer gel dosimeters (PGD) contain radiosensitive chemicals, which, upon irradiation, change their physical density attributing to the underlying process of polymerization and crosslinking. The change in Hounsfield Unit (HU) due to these chemical processes is quantified with 3D imaging and correlated to the dose delivered. Gel dosimeters are particularly beneficial in verifying dosimetric and spatial accuracy of the radiotherapy techniques in which steep dose gradients exist.^12^ Furthermore, to verify geometric precision of a radiotherapy technique with complex spatial arrangements of multiple radiation beam targets, such as LRT, 3D dosimeters can provide definite advantages compared to the established dosimeters used in routine clinical workflow. Therefore, utilizing 3D gel dosimeters for LRT dosimetric verification purposes is a promising solution for a clinical commissioning process.

Standard imaging tools for gel dosimeter readout exist and include magnetic resonance imaging (MRI),^13^, ^14^, ^15^, ^16^, ^17^, ^18^ x‐ray computed tomography (CT),^19^, ^20^, ^21^, ^22^, ^23^, ^24^ and optical‐CT.^25^, ^26^, ^27^, ^28^, ^29^, ^30^, ^31^ A recently studied readout technique, based on Linac‐integrated cone‐beam CT (CBCT), is also used.^27^, ^28^, ^29^, ^30^, ^31^ The advantage of using CBCT as a readout method over the other imaging tools is its superior geometric and spatial accuracy characteristics. Reliance on CBCT for PGD readout method avoids the need to setup the gel dosimeter in a different imaging coordinate (i.e., MRI and x‐ray CT, etc.), therefore, the inherent user setup error for readout is theoretically minimized. Furthermore, an image registration necessary for the gel dosimetry system in the traditional imaging modalities may not be necessary if CBCT is used during the gel analysis. Automatic spatial registration occurs due to the identical DICOM coordinates of the treatment planning system and CBCT imaging. The gel dosimetry workflow with CBCT can provide “actual” geometric accuracy and 3D spatial information, which is highly beneficial in complex radiotherapy techniques such as stereotactic radiosurgery (SRS), stereotactic ablative radiotherapy (SABR), and LRT. Therefore, combining the 3D attributes of the gel dosimeter with the high spatial accuracy of CBCT is a promising solution to verify treatment delivery accuracy.

The work in this research is summarized into three main objectives: (1) To implement an open‐source, reproducible, and semi‐automated vertices placement tool available on GitHub to improve the efficiency of an LRT workflow in the clinic; (2) to generate and evaluate two verification plans on Eclipse v15.6 and pre‐clinical v18.0 by utilizing the LRT software on a gel dosimeter; and (3) to perform a dosimetric verification of the LRT delivery using the polymer gel dosimetry system with CBCT readout, ultimately demonstrating the value of this type of dosimetry system in commissioning complex radiotherapy techniques such as LRT.

## 2 MATERIAL AND METHODS

### 2.1 Automated vertices placement

In this work, an open‐source, reproducible, and semi‐automated software tool (MAAS‐SFRT helper) that is publicly available on the GitHub repository (https://github.com/Varian‐MedicalAffairsAppliedSolutions/MAAS‐SFRThelper) was utilized. The software allows the user to create either regularly shaped (spheres) or irregularly shaped (angles and rods) patterns for the LRT planning. In terms of the sphere lattice structural design, either a hexagonal or rectangular pattern is available. The user specifies the center‐to‐center spacing and the radius of the vertices (spheres) to generate the overall lattice design. The repository was downloaded into the clinical system and the C# code solution was built using the Visual Studio software (Microsoft, Redmond, Washington, USA), though pre‐compiled binaries are also available.

### 2.2 LRT planning

The current study generated the LRT verification plans based on the approach recommended by Grams et al.^32^ Utilizing the MAAS‐SFRThelper software, a hexagonal lattice design with each sphere measuring 1.5 cm in diameter, and a center‐to‐center spacing of 3 cm between the vertices, was generated.^32^ Note that the Grams et al. study recommended a spacing of at least 3 cm between the vertices. The software tool places vertices inside a selected target structure type (GTV, CTV, PTV). In this work, the body contour of the gel phantom symmetrically contracted by 2 cm was used as the GTV (volume: 575.7 cm^3^). The software generated a total of 19 spheres built around the center of the 2 L gel phantom.

The volumetric arc therapy (VMAT) plan consisted of four gantry arcs, consistent with the Grams et al. recommendation of three to four arcs for 10‐25 vertices.^32^ The collimator angles used for each co‐planar gantry arc were 15°, 345°, 75°, 105°. The VMAT optimization was implemented such that 50% of the lattice volume received a prescription dose of 20 Gy.^32^ In order to minimize the valley dose (to < 40% of the maximum peak dose), the 6 MV Flattening Filter Free (FFF) beam was chosen (rather than 10 MV FFF). As per Grams et al., the VMAT optimization was completed once 50% of the volume of each vertex was within ± 50 cGy of the prescription dose on the dose volume histogram (DVH). Eclipse version 15.6 with AcurosXB (Varian Medical Systems, Palo Alto, California, USA) dose calculation algorithm was used to generate the LRT plan. As our cancer center is transitioning to Eclipse v18.0 treatment planning system, the original verification plan was recalculated using the pre‐clinical Eclipse v18.0 for an additional dosimetry check and to evaluate the enhanced leaf model in v18.0.

### 2.3 Gel dosimeter fabrication

A 3D NIPAM polymer gel dosimeter recipe was fabricated based on a previously published protocol.^24^ This recipe comprised of, by weight: 15% N‐isopropylacrylamide (NIPAM, Sigma Aldrich, Mississauga, Ontario, Canada), 4.5% N,N’‐methylenebisacrylamide (BIS or MBA, Sigma), 5% 300 bloom gelatin (Sigma), 10 mM tetrakis(hydroxymethyl)phosphonium chloride (THPC, Sigma), and 75.5% deionized water and the temperature‐controlled mixing procedure was followed according to the protocol.^24^ Once the gel was fabricated, the mixture was transferred into a 2 L jar (Uline, Pleasant Prairie, Wisconsin, USA), cooled in a water bath, and placed in a refrigerator for 5 h.

### 2.4 Gel irradiation, readout, and analysis

**FIGURE 1:**
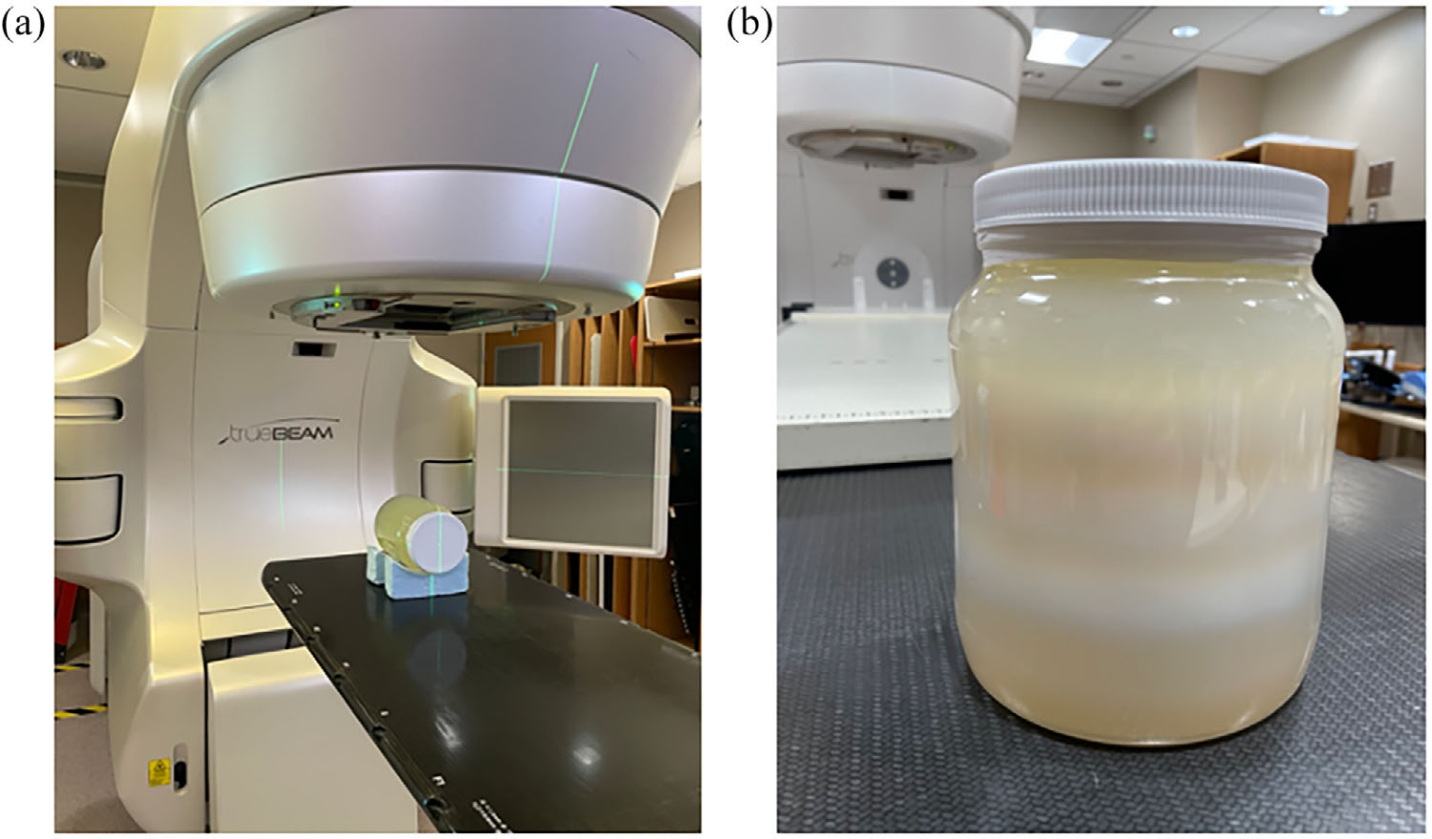
(a) Gel experimental setup. (b) Image of the polymerized gel after irradiation.

A Varian Truebeam Linear accelerator (Varian Medical Systems, Palo Alto, California, USA), with high definition multi‐leaf collimator (HDMLC), was used to deliver the verification plan on the gel dosimeter. The gel dosimeter setup for the experiment is shown in Figure 1a. Figure 1b shows the post‐irradiation density changes (opaque region within the translucent gel) observable on the gel dosimeter.

**FIGURE 2:**
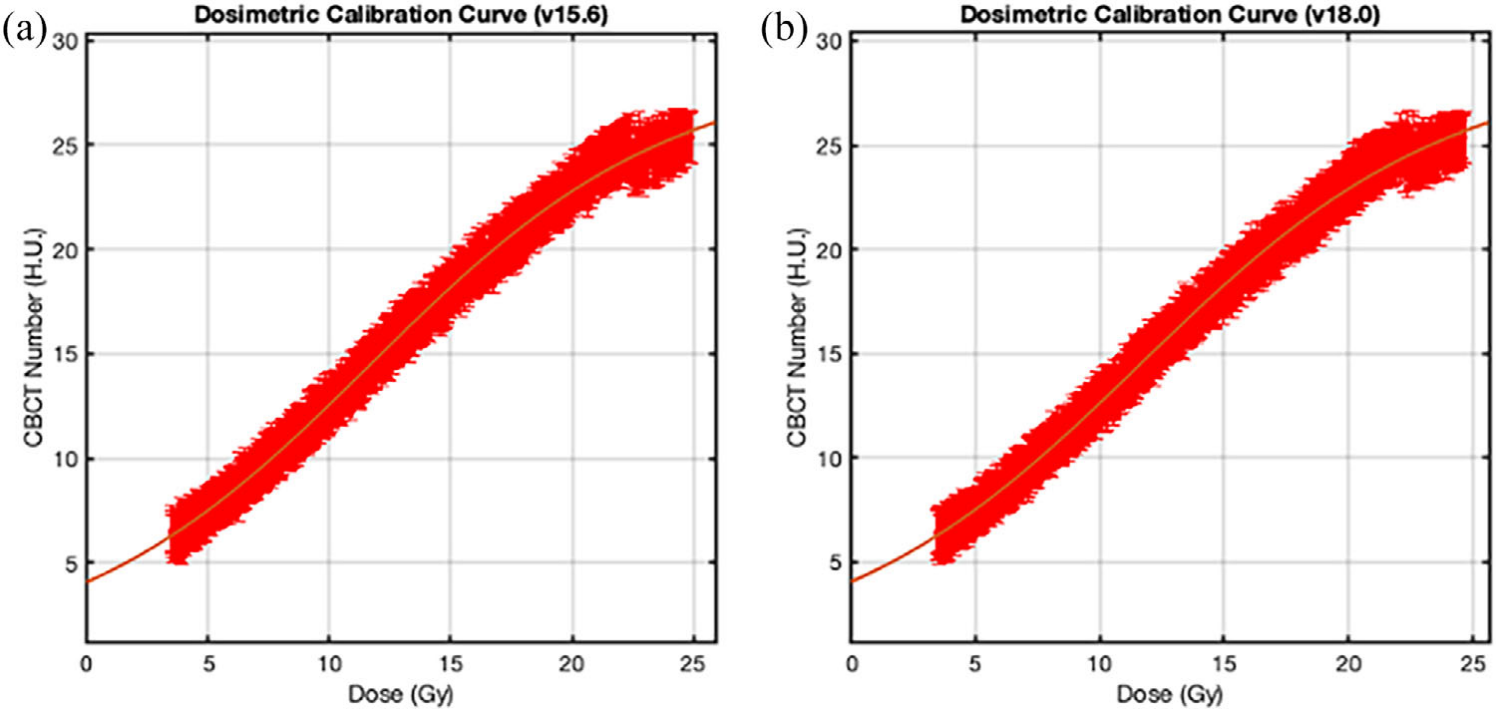
(a) Dosimetric calibration curve obtained from a central slice for Eclipse v15.6. (b) Eclipse v18.0.

Six pre‐irradiation (background) and six post‐irradiation CBCT scans were acquired for the gel dosimetry analysis. The image acquisition parameters of 1836 mAs, 125 kVp, 5400 projections, 0.5 pixel spacing, and 1 mm slice thickness were used to acquire all twelve CBCT images. The background and post‐irradiation images were individually averaged and then subtracted from one another to generate the background subtracted image. An adaptive mean filtering (3 × 3 pixels) and remnant artefact removal (window span = 7) were utilized to reduce image noise and CBCT artefacts on the background subtracted images.^24^, ^33^, ^34^ The central slice from the verification plan that contained the entire range of dose values (0‐24.9 Gy) was used to self‐calibrate the image by a pixel‐by‐pixel calibration technique with the corresponding gel image slice. The dose‐response curve (Equation 1, Figure 2a, b) was then used to calibrate the entire gel image volume. Note that the gel dosimeter was calibrated separately for each of the Eclipse v15.6 and v18.0 verification plans.

The equation of the dosimetric calibration curve is presented below:

(1) $$\Delta N_{\mathrm{CBCT}}=\alpha +\beta \;tanh\left(\gamma D-\varphi \right)$$

where Δ*N_CBCT_* is the change in CBCT number of the gel (background subtracted image), *D* represents dose in Gy, α,β,γ,φ are the parameters of the equation. The parameters defining the dosimetric calibration curve, acquired from the two plans with 95% confidence interval, are provided in Appendix A (Table A1).

## 3 RESULTS

**FIGURE 3:**
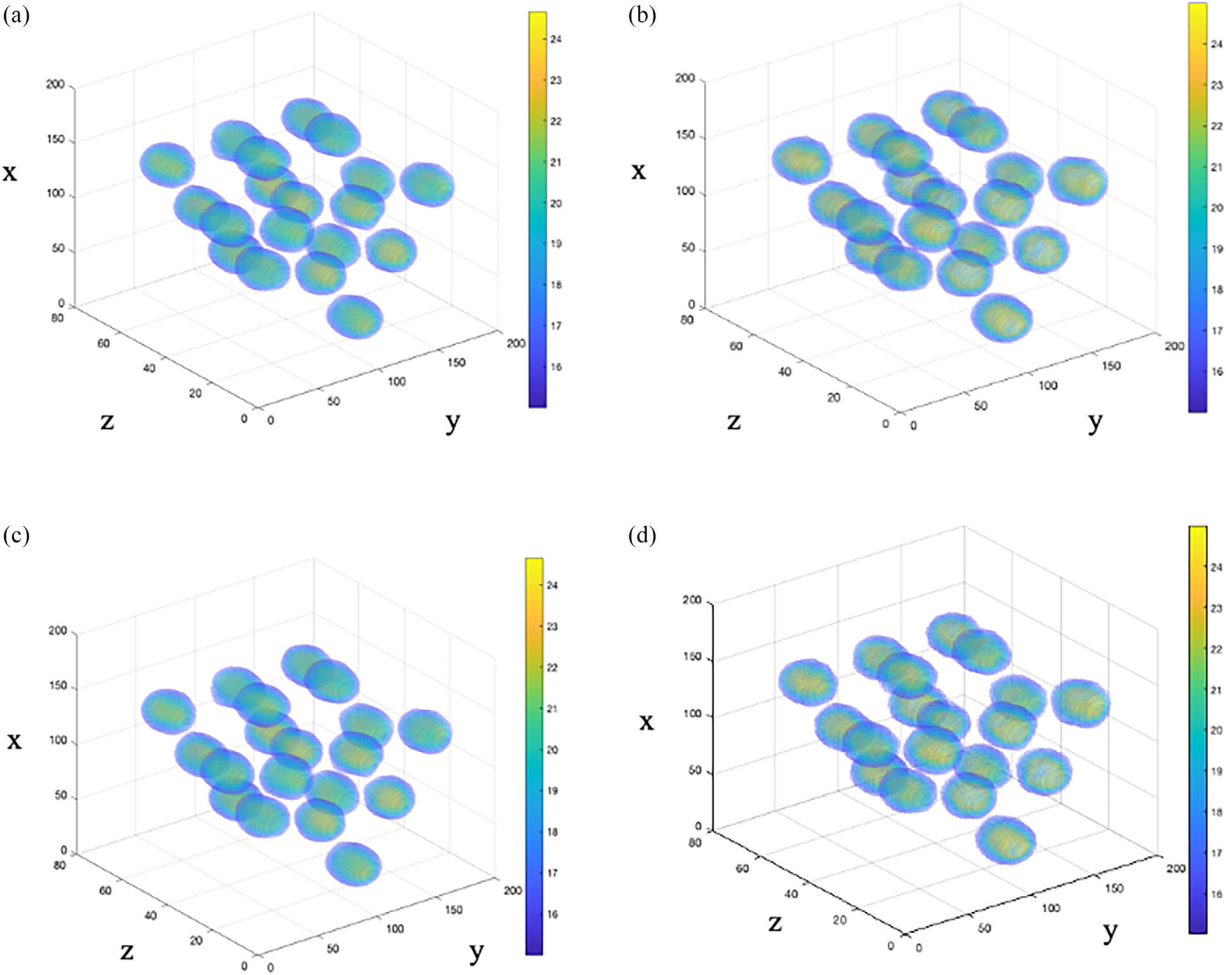
(a) The 3D visualization of Eclipse v15.6 plan. (b) Calibrated gel using v15.6 plan. (c) Eclipse v18.0. (d) Calibrated gel using v18.0 plan.

Figure 3 illustrates a 3D visualization of the 19 spheres in the image volume of (a) Eclipse v15.6 plan and (b) the corresponding calibrated gel dosimeter; (c) v18.0 plan and (d) the corresponding calibrated gel dosimeter, displayed on the same colormap. The figure presents the spatial localization of the 19 spheres on the same axis. Qualitatively, the spatial positions of the vertices in the measured gel appear consistent respective to the Eclipse plan. Note that Table 1 presents the quantitative information regarding the dimensions of the spheres.

**TABLE 1:** The equivalent diameter of the isodose volume of the prescription dose (20 Gy) in Eclipse v15.6 plan, Eclipse v18.0 plan, and respective Gel image volume.

	Eclipse v15.6 plan (Equivalent diameter in mm)	Gel v15.6 (Equivalent diameter in mm) ± Normalized difference between the gel and plan (mm)	Eclipse v18.0 plan (Equivalent diameter in mm)	Gel v18.0 (Equivalent diameter in mm) ± Normalized difference between the gel and plan (mm)
Sphere 1	13.0	14.3±0.10	12.7	14.2±0.12
Sphere 2	12.6	13.8±0.10	12.6	14.3±0.13
Sphere 3	12.9	14.0±0.08	12.7	13.9±0.09
Sphere 4	13.0	13.9±0.10	12.2	13.6±0.11
Sphere 5	12.8	14.4±0.13	12.7	13.5±0.06
Sphere 6	12.9	13.6±0.05	12.6	13.7±0.09
Sphere 7	12.4	12.9±0.04	12.1	12.7±0.05
Sphere 8	12.3	12.7±0.03	12.1	12.2±0.01
Sphere 9	12.4	12.9±0.04	12.1	12.7±0.05
Sphere 10	12.6	12.4±0.01	12.3	12.5±0.02
Sphere 11	12.3	13.0±0.06	12.1	12.9±0.07
Sphere 12	12.6	12.9±0.02	12.4	12.7±0.02
Sphere 13	12.5	11.1±0.11	12.3	10.9±0.11
Sphere 14	12.9	13.5±0.05	12.5	13.3±0.06
Sphere 15	12.4	13.2±0.06	12.1	13.0±0.07
Sphere 16	12.3	13.1±0.07	12.0	13.0±0.08
Sphere 17	12.6	13.4±0.06	12.2	13.2±0.08
Sphere 18	12.9	13.7±0.06	12.6	13.5±0.07
Sphere 19	12.3	13.4±0.09	12.0	13.3±0.12
Average±SD	12.6	13.3±0.2mm	12.3	13.1±0.2mm

The error was calculated based on the normalized difference between the Eclipse plan and the gel measurement.

**FIGURE 4:**
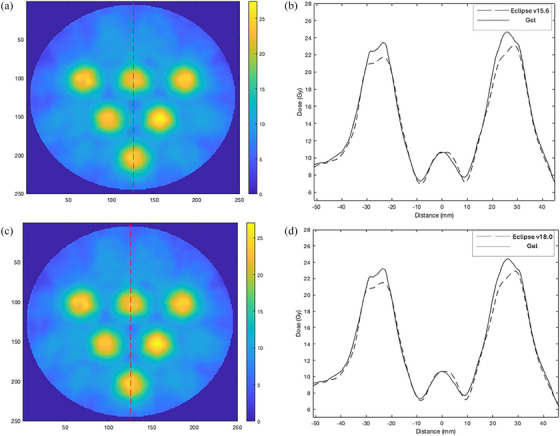
(a) and (c) demonstrate a line profile through a 2D slice, (b) dose profile along the line in transverse plane (anterior‐posterior) for Eclipse v15.6, (d) Eclipse v18.0.

Figure 4 shows (a) and (c) vertical line profiles through the Eclipse v15.6 and v18.0 calibrated gel slices and (b) the corresponding dose profiles obtained through a transverse plane (anterior‐posterior) of both Eclipse v15.6 plan and gel dosimeter, (d) Eclipse v18.0 and gel dosimeter. The overall dose profile (location of the peak and valley dose) appeared consistent between the plan and measured dose distribution. The peak dose at the center of the two vertices was about 10% higher in the measured gel compared to the Eclipse plan. The width of the profile at the 20 Gy prescription dose showed good consistency between the plans and measurements. In v15.6 experiment, a valley dose of 29.3% was observed in this slice (both in Eclipse plan and the measured gel). In v18.0 experiment, a valley dose of 29.5% was measured at the same pixel location.

**FIGURE 5:**
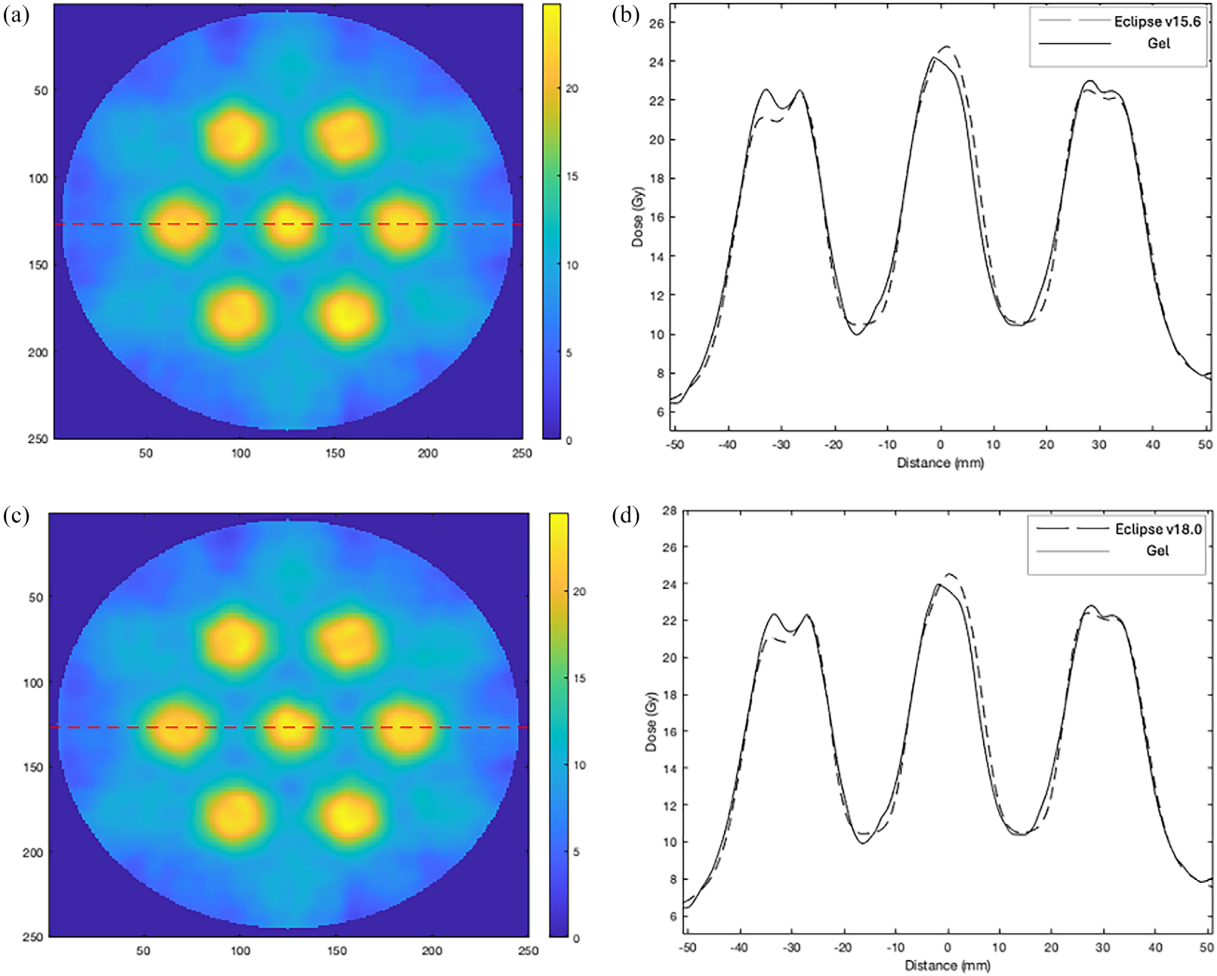
(a) and (c) demonstrate a line profile through a 2D slice, (b) dose profile along the line in transverse plane (left‐right) for Eclipse v15.6. (d) Eclipse v18.0.

In Figure 5, a similar analysis is shown. Figure 5a and c shows the horizontal line profiles in the gel slices, and Figure 5b and d present the corresponding dose profiles in Eclipse plan v15.6 and v18.0 with the gel measurements, respectively. The maximum peak dose in the Eclipse plan and gel dosimeter appeared more consistent in this case compared to Figure 4. The width of the profile across the 20 Gy dose line appeared consistent between the Eclipse plan and the gel dosimeter. In v15.6, a valley dose of 39.9% was measured in this slice, and a slightly lower valley dose was observed in the gel compared to Eclipse plan (42%) in certain areas of the dose profile. In v18.0 calibrated gel dosimeter, a valley dose of 40.2% was measured at the same pixel location.

**FIGURE 6:**
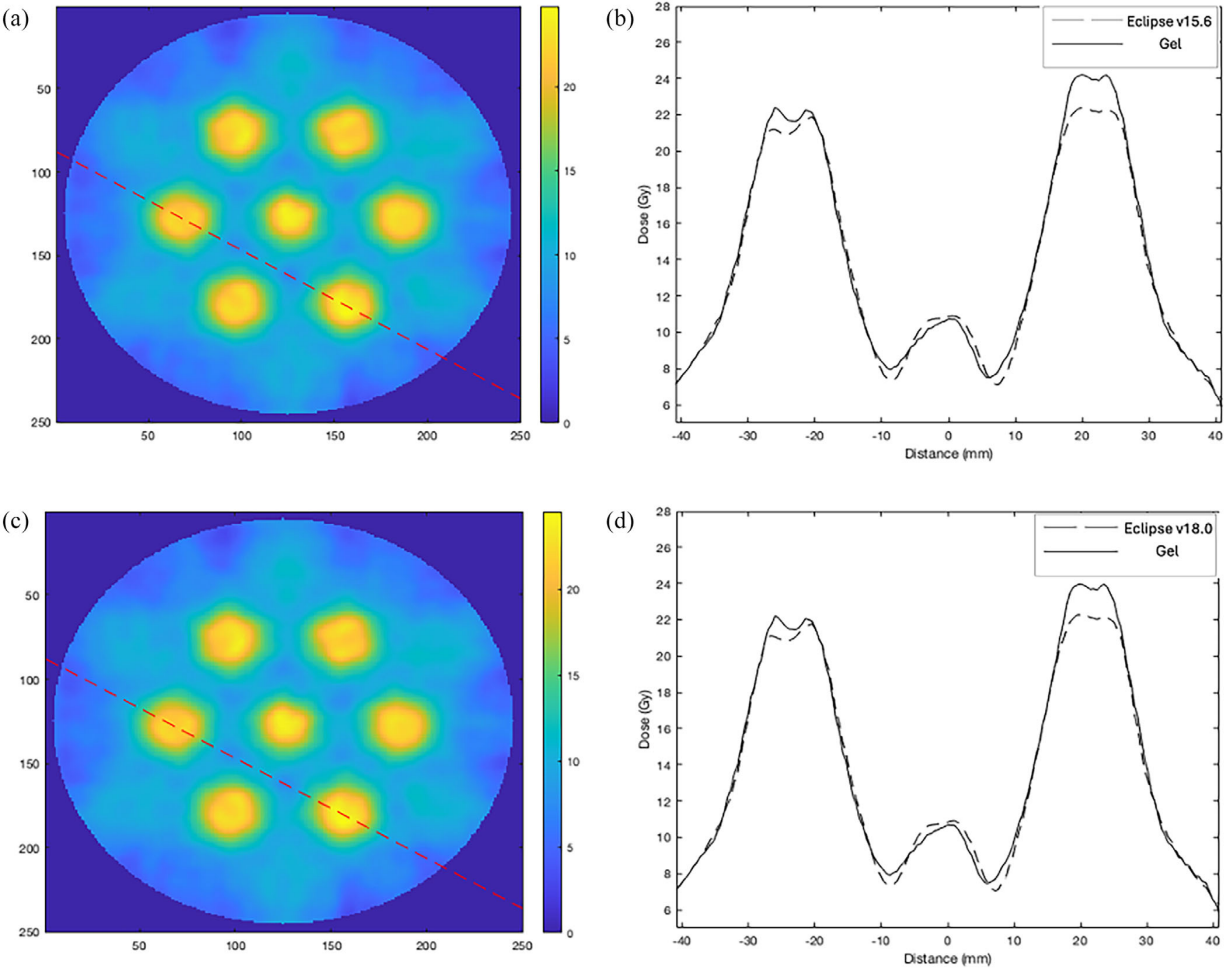
(a) and (c) demonstrate a diagonal profile through a 2D slice, (b) dose profile in transverse plane for Eclipse v15.6, and (d) Eclipse v18.0.

Figure 6a and c present a diagonal profile through the two targets and local minimum points. Figure 6b and d shows the corresponding dose profiles for both Eclipse v15.6 plan and v18.0 plan with the measured gel profile. The width of the profile along the 20 Gy prescription mark was consistent in the two periphery targets and slightly less so in the central target. The low valley dose measured 30.1% and 30.3% for gels with v15.6 and with v18.0, respectively.

**FIGURE 7:**
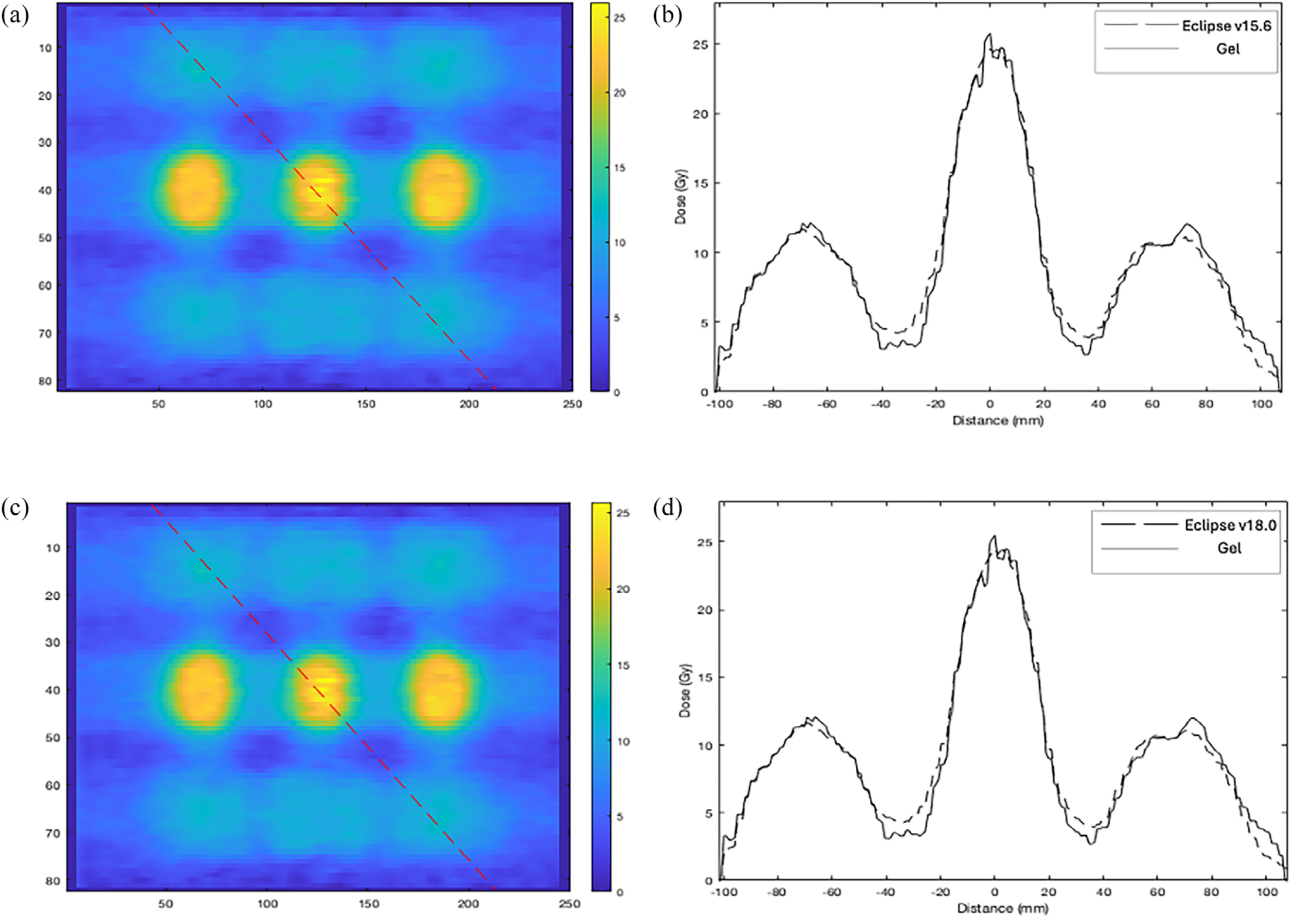
(a) A line profile through a 2D slice, (b) dose profile along the line in coronal plane (superior‐inferior) in v15.6, (c) v18.0 calibrated gel, and (d) dose profile through v18.0 plan and gel measurement. Note that the spherical region appears oblong because the LRT plan utilized here are co‐planar arcs. Therefore, a sharp cutoff in sup‐inf direction is observed in the figure.

Figure 7a and c illustrate the dose profile diagonally, along a sagittal view of the gel slice plotted through the superior‐inferior direction. Figure 7b shows the corresponding dose profile of the Eclipse plan v15.6 and the gel measurement and 7d shows the profile along v18.0 plan and its calibrated gel measurement. Both the peak and valley dose of the gel match well with the respective Eclipse plans. In v15.6 plan, a valley dose of slightly more than 15.5% was observed in this slice, however, the valley dose in measured gel (10.7%) appeared lower than the Eclipse plan dose. In v18.0 calibrated gel image, the valley dose measured 10.9%, slightly higher than the v15.6 calibrated gel.

**FIGURE 8:**
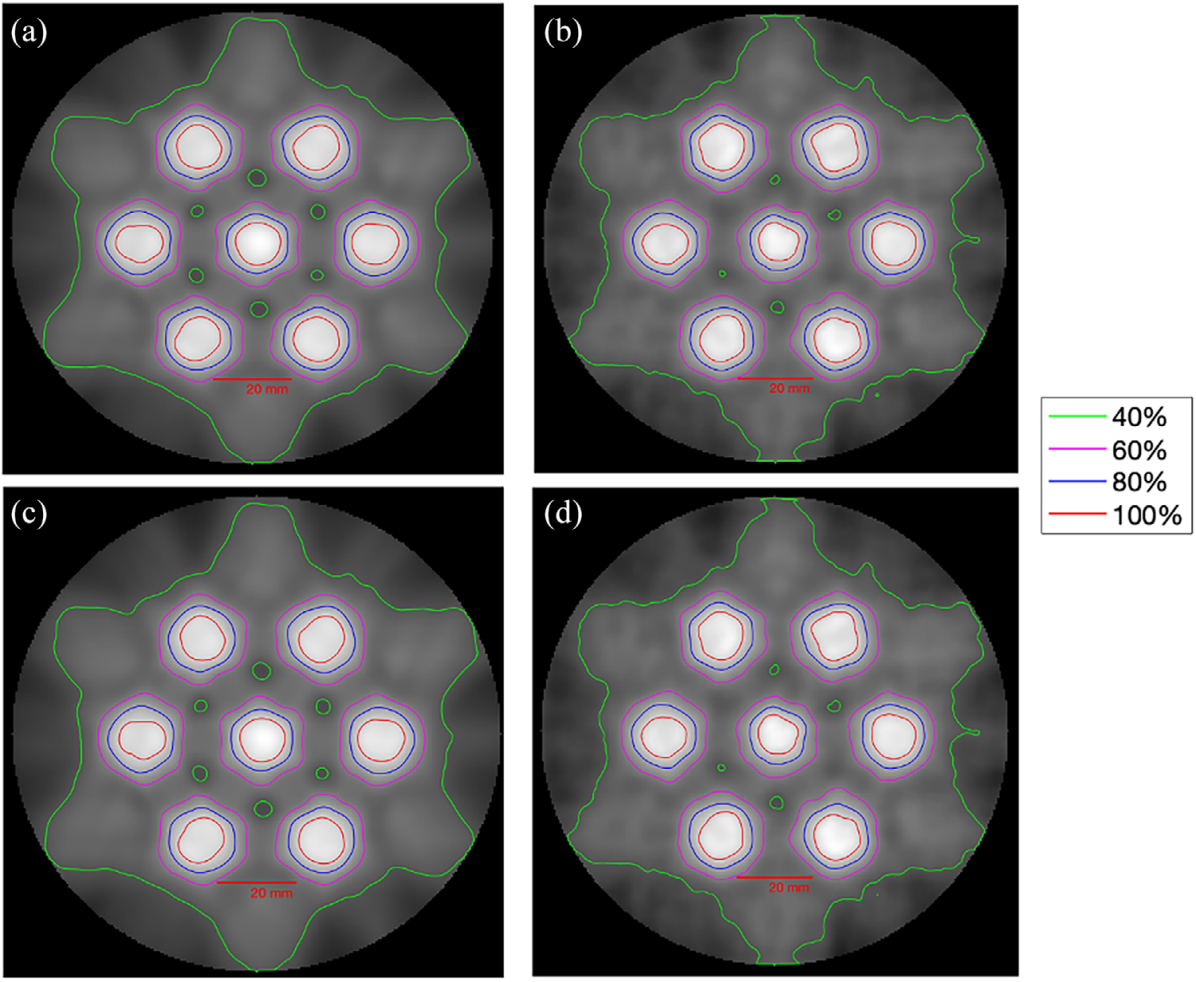
(a) The 2D isodose map of a central slice of the Eclipse v15.6 plan. (b) Calibrated Gel (v15.6), (c) v18.0 plan, (d) Calibrated Gel (v18.0).

Figure 8 presents an image of the of 40%–100% isodose lines with 20% increment in the Eclipse v15.6 plan (a) and its corresponding gel slice (b), Eclipse v18.0 plan (c), and its corresponding gel slice (d). The 100% isodose line represents the prescription dose of 20 Gy since both the images were normalized to the prescription dose. Qualitatively, the 100% isodose line appeared consistent between the plan and the measured gel. A low valley dose (dark spots) between the high‐dose spheres, at the center of the image in Figure 8a and 8c were apparent, at least in the planning images and less visible in the gel images.

The equivalent diameter of 20 Gy isodose volume for each of the vertices were calculated. Table 1 presents the equivalent diameter of the 100% isodose volume (refer Figure 8 for qualitative comparison) of both the Eclipse v15.6 plan and the gel dosimeter, and Eclipse v18.0 plan and gel dosimeter respectively. The average diameter resulted in 12.6±0.3mm for Eclipse v15.6 plan, 12.3±0.3mm for Eclipse v18.0 plan. A diameter of 13.3±0.2mm (gel volume ± standard deviation) was measured for the v15.6, and 13.1±0.2mm for the v18.0, for the 19 spheres. The error for each measurement was calculated by obtaining the difference between the Eclipse plan and gel measurement, normalized to the measured plan diameter value.

**FIGURE 9:**
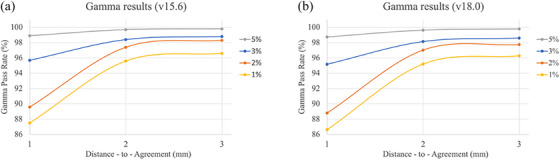
(a) The scatter plot of the 3D global gamma pass percent as a function of distance‐to‐agreement in mm for Eclipse v15.6 and (b) v18.0 experiment.

Figure 9 reports the 3D global gamma analysis utilizing 12 commonly used gamma criteria with 10% threshold: the 3D gamma pass percent are all above 95% except the 2% 1 mm and 1% 1 mm criteria. Figure 8 presents the gamma pass percent plotted for each dose difference criteria (5%, 3%, 2%, and 1%) as a function of distance to agreement in mm.

## 4 DISCUSSION

The current study demonstrated the efficacy of a publicly available semi‐automated LRT planning process, which comprised of an open‐source, reproducible, and automated vertices placement tool. This code was built within the Eclipse Scripting Applications Programming Interface (ESAPI) and was easily integrated into the Eclipse treatment planning workflow. Moreover, polymer gel dosimetry system was utilized to verify the dosimetric accuracy of the planned LRT delivery. To the best of our knowledge, this novel application of 3D gel dosimetry utilizing CBCT to verify a LRT technique is the first to be described.

Recently, there have been publications demonstrating a fully automated LRT planning process. A study by Gaudreault et al. published in 2023 proposed a completely automated LRT workflow into the commercial treatment planning system that generated vertices as well as an optimized treatment plan. The automated contouring approach in the study was achieved by initially placing the vertex at the center of an “updated” gross tumor volume (original GTV contour contracted isotopically by a 5 mm margin), and then build the rest of the vertex around the first vertex in a layer‐by‐layer fashion. The work reported that the dosimetry check from the fully automated treatment planning was comparable to the manual LRT planning approach.^11^ Their study provided an innovative and comprehensive solution for LRT planning without any manual intervention in both the treatment planning and optimization. Another recent study published in the same year by Zhang et al., proposed an innovative vertices placement optimization method to improve peak‐valley‐to‐dose ratio and organ at risk sparing.^35^ The vertices placement in the study were optimized based on the two optimization objectives: peak‐valley‐to‐dose ratio and organ at risk sparing. The study introduced a novel planning process in which both the vertices placement and photon/proton plan variables (photon fluence and proton spot weights) were optimized to generate a treatment plan. A study by Deufel et al. utilized a Monte Carlo‐based algorithm to design and automatically place the vertices inside a GTV. The study concluded that their approach yields similar dose metrics, increases inter‐planar consistency for large tumor targets, and minimizes the usage of clinical resources compared to a manual planning approach.^36^

The vertices placement approach employed in our study places the vertices in a hexagonal closed packed (HCP) structure, where each sphere in the center is linked with 12 neighboring spheres. A x (mm) and y (mm) shift can be applied in order to move the entire lattice structure around the tumor volume based on the preference of the user. Although the overall lattice placement was not optimized in our study, the valley dose is around 10%‐40% of the maximum dose in both the Eclipse plan and measured gel. In the sagittal view, a local minimum of 10.7% and 10.9% was observed in gel calibrated with Eclipse v15.6 and v18.0, respectively. Therefore, the verification plan on the 2 L gel phantom was concluded to have reached the optimization objectives according to the guidelines recommended by Grams et al.^32^ One improvement that can be made in our LRT planning approach is to incorporate an optimization process in the lattice placement and the overall structural design to further improve the verification plan.

One of the challenges in implementing LRT in clinic is the lack of standardized guidelines for LRT treatment planning including lattice array design, optimal number of vertices, spatial location, and the dimensions of the spheres.^5^ Some studies reported an arbitrary lattice design based on the radiation oncologist and medical physicist's recommendation, founded on the size and proximity of the location of the tumor to organ at risks (OARs).^7^, ^37^, ^38^, ^39^ Another LRT efficacy case study utilized an in‐house script to optimize the number of vertices in a tumor volume.^40^ Despite the lack of consensus in LRT planning approach, removal of vertices closer than 1.5 cm to the OARs is consistently observed.^5^

The Eclipse v15.6 beam model used in our study was comprehensively commissioned with cc13 ionization chamber (IBA Dosimetry Group, Herndon, Virginia, USA), microDiamond detector (PTW, Freiburg, Germany), GafChromic film (Ashland Advanced Materials, Bridgewater, New Jersey, USA), and ArcCHECK (Sun Nuclear, Melbourne, Florida, USA). Furthermore, our daily patient‐specific quality assurance is typically done with Electronic Portal Imaging Device‐based dosimetry tool. The v15.6 beam model has been in clinical use for the last several years treating various patients with stereotactic radiosurgery and stereotactic ablative radiotherapy techniques on a daily basis. Therefore, we implemented an additional 3D gel dosimetry tool to verify the calculation and delivery of the LRT technique with the clinically utilized beam model. However, clinical implementation of v18.0 would require comprehensive dosimetric verification with independent tools as described above.

Our study demonstrated a novel application of 3D gel dosimetry in verifying the LRT delivery. A qualitative comparison between the Eclipse planned dose and measured gel dose were consistent in the 1D dose profiles and 2D isodose map (Figures 4, 5, 6, 7, 8). Utilizing a gel dosimetry system in this case provided an advantage over the traditional dosimeter, since an entire 3D dose information was efficiently acquired within a single experiment. The equivalent diameter of the 20 Gy isodose volume in both Eclipse plan and measure gel was also measured. The mean diameters were 12.6±0.3 and 13.3±0.2mm for Eclipse v15.6 plan and gel, respectively. For v18.0 experiment, a diameter of 12.3±0.3 and 13.1±0.2mm was measured for the plan and gel, respectively. The VMAT optimization protocol by the Mayo clinic study concluded that the original 15 mm diameter vertex should contract to an approximate size of 1 cm,^32^ although the study has not provided a stringent quantitative measurement of the value. Figure 3 shows a 3D visualization achievable with the gel dosimetry system. The placement of the vertex in the Eclipse plan and measured gel appeared consistent in terms of a qualitative analysis, however the gel image volume appeared to have a slightly higher maximum dose in the entire dose distribution. The 3D global gamma pass rates were more than 95% for all gamma criteria were obtained, except the 2% 1 mm and 1% 1 mm criteria. Thus, with all the analyses combined, the study demonstrated that utilizing a gel dosimeter in verifying LRT delivery provided a comprehensive evaluation of the treatment delivery process within a single gel irradiation.

## 5 CONCLUSION

In order to achieve widescale clinical adoption of the emerging LRT technique, two key challenges must be addressed: Efficient LRT treatment planning and dosimetric verification. In this work, a semi‐automated vertex placement tool was used to increase the efficiency of the LRT planning process. The optimization of the VMAT plan was completed according to the guidelines given by Grams et al.^32^ The delivery of this complex dose distribution was verified with a novel 3D polymer gel dosimetry system using CBCT readout, achieving excellent 3D gamma results. Consequently, this work has demonstrated the application of polymer gel dosimetry as an invaluable tool for the commissioning of this complex radiotherapy technique.

## AUTHOR CONTRIBUTIONS

**Tenzin Kunkyab**: Project lead; treatment planning; performed experiments/data collection; methodology; interpretation; analysis; project administration; manuscript writing. **Anthony Magliari**: Guidance in LRT planning; manuscript revision. **Andrew Jirasek**: Methodology; interpretation; analysis; funding acquisition; manuscript revision. **Benjamin Mou**: Conceptualization; clinical guidance; manuscript revision. **Derek Hyde**: Conceptualization; treatment planning; funding acquisition; interpretation; manuscript revision; supervising the author.

## CONFLICT OF INTEREST STATEMENT

None except Anthony is employed by Varian.

## APPENDIX A

Table A1

**TABLE A1:** The calibration curve parameters.

Calibration parameters	α	β	γ	φ
Eclipse v15.6	14.5 (14.4,14.6)	14.2 (13.9,14.4)	0.08 (0.07,0.08)	0.94 (0.92,0.98)
Eclipse v18.0	14.5 (14.5,14.7)	14.2 (14.0,14.5)	0.08 (0.07,0.08)	0.95 (0.92,0.97)
